# Psychometric testing of the British English Workplace Activity Limitations Scale in four rheumatic and musculoskeletal conditions

**DOI:** 10.1093/rap/rkad028

**Published:** 2023-03-10

**Authors:** Alison Hammond, Alan Tennant, Angela Ching, Jennifer Parker, Yeliz Prior, Monique A M Gignac, Suzanne M M Verstappen, Rachel O’Brien

**Affiliations:** Centre for Human Movement and Rehabilitation, School of Health and Society, University of Salford, Salford, UK; Leeds Institute of Rheumatic and Musculoskeletal Medicine, University of Leeds, Leeds, UK; Centre for Human Movement and Rehabilitation, School of Health and Society, University of Salford, Salford, UK; Centre for Human Movement and Rehabilitation, School of Health and Society, University of Salford, Salford, UK; Centre for Human Movement and Rehabilitation, School of Health and Society, University of Salford, Salford, UK; Institute of Work and Health, Toronto, ON, Canada; Dalla Lana School of Public Health, University of Toronto, Toronto, ON, Canada; Centre for Epidemiology Versus Arthritis, Centre for Musculoskeletal Research, Faculty of Biology, Medicine and Health, University of Manchester, Manchester, UK; Manchester Academic Health Science Centre, Manchester, UK; NIHR Manchester Biomedical Research Centre, Manchester University NHS Foundation Trust, Manchester, UK; College of Health, Well Being and Life Sciences, Sheffield Hallam University, Sheffield, UK

**Keywords:** patient-reported outcomes, work, work rehabilitation, arthritis, musculoskeletal, rehabilitation

## Abstract

**Objectives:**

The aims were to validate a British English version of the Workplace Activity Limitations Scale (WALS) linguistically, then test this psychometrically in RA, axial spondyloarthritis (axSpA), OA and FM.

**Methods:**

The WALS was forward translated, reviewed by an expert panel, and cognitive debriefing interviews were conducted. Participants completed a postal questionnaire booklet. Construct (structural) validity was examined by fit to the Rasch measurement model. Concurrent validity included testing between the WALS and the Work Limitations Questionnaire-25 (WLQ-25). Two weeks later, participants were mailed a second questionnaire booklet for test–retest reliability.

**Results:**

Minor wording changes were made to the WALS, then 831 employed participants completed questionnaires: 267 men and 564 women; 53.5 (s.d. 8.9) years of age; with condition duration 7.7 (s.d. 8.0) years. The WALS satisfied Rasch model requirements, and a WALS Rasch transformation table was created. Concurrent validity was strong with the WLQ-25 (RA *r*_s_ = 0.78; axSpA *r*_s_ = 0.83; OA *r*_s_ = 0.63; FM *r*_s_ = 0.64). Internal consistency was consistent with group use (α = 0.80–0.87). Test–retest reliability was excellent, with intraclass correlation coefficient (2,1) at ≥0.90.

**Conclusion:**

A reliable, valid British English version of the WALS is now available for use in the UK.

Key messages

- WALS content is considered highly relevant by working people with RA, OA, axSpA and FM.
- WALS has good reliability and construct (structural), concurrent and discriminant validity.
- WALS can be used to evaluate work difficulties, need for work rehabilitation and treatment outcomes.

## Introduction

Work participation (i.e. paid work) is important for the health and well-being of people with rheumatic and musculoskeletal disorders (RMDs). Yet they have a shorter healthy working life expectancy [1] and are less likely to be employed compared with those without long-term health conditions [2]. Working people with RMDs can struggle to manage work, leading to presenteeism (i.e. reduced at-work productivity owing to health problems [3]). This is an important target for improvement in medical, rehabilitation and vocational interventions, and from the perspectives of people with RMDs [4]. Outcome measures assessing at-work productivity, tested across a range of RMDs, can help to direct and evaluate such interventions.

The OMERACT Work Productivity Group identified two patient-reported outcome measures of at-work productivity suitable for use in RMD [5, 6]. The Work Limitations Questionnaire-25 (WLQ-25) measures duration of difficulty with work activities (work productivity) [7]; and the Workplace Activity Limitations Scale (WALS) measures the amount of difficulty with work activities (work ability) [4, 8]. People with RA and OA preferred the WALS over the WLQ-25 as an outcome measure [9].

The WALS was developed and tested psychometrically in Canada and has been used there in studies in inflammatory arthritis [i.e. RA, PsA and axial spondyloarthritis (axSpA)], OA, lupus and scleroderma [10–15]. In RA and OA, it has the following characteristics: good content validity, comprehensibility and content relevance [9]; low respondent burden [16]; and concurrent validity with other work measures [6, 17]; although there is only limited evidence for its test–retest reliability [6]. It has potential for clinical and research use in the UK. The WALS was developed in Canadian English. Before use in the UK, it should be validated linguistically (i.e. translated and culturally adapted) into British English (a different form of the same language), then tested psychometrically [18]. Although most Canadian English is understandable in the UK, some words used in the WALS have different meanings, e.g. ‘subway’ means a rapid transport system in North America but an underpass for crossing roads in the UK. The aims of the present study were therefore as follows: to validate linguistically, investigate content validity, and evaluate the psychometrics of a British English WALS among employed people with RA, axSpA, lower limb OA and FM in the UK. Testing should include both classical testing and item response theory (e.g. Rasch analysis) to establish psychometric properties (e.g. reliability and validity) [19].

## Methods

The study design used cross-cultural adaptation, followed by cross-sectional surveys to establish psychometric properties of the WALS. The COnsensus‐based Standards for the selection of health Measurement Instruments (COSMIN) checklists were followed [19, 20]. Ethical approval was obtained from the National Research Ethics Service Committee East Midlands, Leicester South (17/EM/0409). All participants provided written, informed consent.

### Participants and recruitment

Patients were recruited from 41 secondary care and six community National Health Service Trusts’ Rheumatology, Orthopaedic or Therapy outpatient clinics, with some participants from our research group’s Arthritis Volunteer Register, in the UK. Participants were eligible if they were: ≥18 years of age, in paid employment ≥1 day per week, currently working or on <4 weeks sick leave, with participation delayed until at work, and had a primary diagnosis of RA or undifferentiated inflammatory arthritis (UIA), axSpA, OA (knee and/or hip) or FM. Diagnoses were confirmed by a rheumatologist for RA, UIA and axSpA or by a rheumatologist, orthopaedic surgeon, general practitioner or extended scope physiotherapist for OA and FM. Participants needed to be able to read, write and understand British English and were ineligible if on long-term sick leave, because they were unable to complete the work measures. Patients were identified by research facilitators or therapists using these criteria and given a short study explanation and information pack. The latter included a reply form, including diagnosis, employment and sick leave status, to check eligibility criteria.

### Data collection

In phase 1, linguistic validation and cross-cultural adaptation were conducted to ensure that the wording in the WALS was considered comprehensible by participants. Content validity (i.e. the degree to which the content of a patient*-*reported outcome measure is considered an adequate reflection of what is being measured) was also tested [18, 21] (see Supplementary Data S1, available at *Rheumatology Advances in Practice* online).

In phase 2, for psychometric testing, participants were mailed a paper questionnaire booklet to complete at home [test 1 (T1)]. Two weeks after return of the questionnaire, they were mailed a second questionnaire [test 2 (T2)], to assess test–retest reliability. Participants were sent a reminder letter after 2 weeks, followed at 4 weeks by another reminder and questionnaire booklet, if needed.

The T1 booklet included demographic data: age, sex, living arrangements, education status, condition duration, medication regimen, employment status and job title, to allow coding to job skill level {1 = elementary occupations; 2 = requiring compulsory education*/*work-related training; 3 = post-compulsory education (sub-degree) or longer work experience; 4 = degree education or equivalent experience [22]}.

The T1 booklet also included the British English WALS, consisting of 12 items, measured on a scale of 0–3 for difficulty in performing work activities (0 = no difficulty; 3 = unable to do; Supplementary Data S2*,* available at *Rheumatology Advances in Practice* online). The WALS includes: eight physical activity items; three about managing work; and concentration at work [12]. The instructions state that respondents should answer about their work performance without help from others or using special gadgets or equipment, in order that their answers are not confounded by the use of workplace behavioural coping strategies [10]. The recall period is not specified. Those items answered as ‘not applicable to my job’ are scored 0. Scoring allows up to three missing items, which can be imputed using the individual’s mean or median scores (depending on the data distribution). A summed score is calculated (0–36), with scores ≥9 being associated with greater absenteeism, job disruptions and need for work accommodations, compared with those scoring <5 [13].

To test concurrent validity, several work and health measures were included in the T1 questionnaire booklet. Some of these were condition-specific measures, and therefore four separate condition-specific T1 questionnaire booklets were used, with participants completing the booklet relevant for their condition. For all measures, a higher score indicates worse status. Three work measures were included. The WLQ-25 consists of 25 items in four subscales (1–5 scale), indicating the percentage time in the past 2 weeks that physical work, time, mental–interpersonal and output demands were limited [7]. From these, the WLQ Percentage Productivity Loss [7] and Summed scores [23] are created. Two forms of the Work Instability Scale (WIS) were used: the RA-WIS was included in those questionnaires for people with RA, OA or FM, and the AS-WIS for axSpA [24–26]. Both forms measure the degree of mismatch between the respondent’s work abilities and their job demands. The RA-WIS includes 23 true/false items and the AS-WIS 20 items. Both have cut-points indicating low, moderate and high work instability (RA-WIS <10 and >17; AS-WIS <11 and >18). The third work measure was the Work Productivity and Activity Impairment (WPAI) (General Health) scale, which includes six items, from which a Percentage overall work impairment due to health (in the past 7 days) score is calculated [27].

For RA, the condition-specific health measures included in the T1 booklet were: the Rheumatoid Arthritis Impact of Disease (RAID) scale, consisting of seven 0–10 numerical rating scales (NRS; e.g. pain, fatigue, function) scored by summing weighted NRS scores [28]; and the HAQ, consisting of 20 daily activities rated 0–3 (0 = not at all difficult; 3 = unable to do) [29]. The HAQ was scored by summing all items (0–20 = mild; 21–40 = moderate; 41–60 = severe disability) without adjustment for using aids and devices [30]. For axSpA, the health measures were: the BASDAI, in which the average score (0–10) is calculated from six 10 cm visual analogue scales (VAS) of symptom severity (e.g. fatigue, spinal pain [31]); and the BASFI, in which an average score (0–10) is calculated from ten 10 cm VAS of physical function [32]. For OA, two subscales of the WOMAC were included [pain (five items) and physical function (17 items); both scored from 0 = none to 4 = extreme], with total scores for each subscale calculated [33]. Finally, for FM, the Revised Fibromyalgia Impact Questionnaire (FIQR) was included. This consists of three subscales rated on 0–10 NRS [overall impact (two items); symptoms (10 items); and function (nine items)]. Subscale and overall total scores were then calculated [34]. For all four conditions, an additional health question was about perceived health status: ‘Considering all the ways that your condition affects you, how have you been over the past month?’ (scored from 1 = very good to 5 = very poor).

At test 2, participants completed the WALS, perceived heath status and also an item on perceived change in health status: ‘Overall, how much is your arthritis*/*condition troubling you now compared with when you last completed this questionnaire?’ (1 = much less; 3 = about the same; 5 = much more).

### Sample size

Given that Rasch analysis was used to assess construct (structural) validity, a minimum of 150 cases are needed within each condition group [35]. We aimed to collect *≤*250 to ensure a broad spread of responses. At least 79 sets of repeated responses were needed to demonstrate that a test–retest correlation of 0.7 differed from a background correlation (constant) of 0.45, with 90% power at the 1% significance level. A test–retest reliability correlation of 0.7 is considered a minimum acceptable level [36].

### Statistical analyses

Demographic, work and health measures were summarized descriptively, as appropriate. RUMM 2030+ software was used for Rasch analysis [37]. Given that all work and health measures either consisted of ordinal data or were not normally distributed, non-parametric statistical tests were conducted using the Statistical Package for the Social Sciences (SPSS) v.26 [38]. The following psychometric properties were assessed.

#### Compliance

Compliance (i.e. the amount of missing data) was assessed by identifying the number (percentage) of missing data items and also WALS which could not be scored.

#### Validity

Construct (structural) validity measures the degree to which the scores of a patient*-*reported outcome measure adequately reflect the dimensionality of the construct being measured (e.g. do all scale items measure the same construct, and are items independent of one another?). The first analytical strategy was testing the fit of the WALS for each condition to the Rasch measurement model [39]. The approach also tested cross-diagnostic validity to test for invariance (i.e. whether the scale can be used to assess group differences because items are being interpreted similarly across groups; e.g. across conditions, age groups and sex). For interested readers, full details of the analysis are given in Supplementary Data S3 and Table S1, available at *Rheumatology Advances in Practice* online, and described in detail elsewhere [40].

Concurrent validity (i.e. the degree to which scores are consistent with hypotheses, e.g. that scores on other relevant measures are correlated with the WALS) was assessed using Spearman’s correlations with work and health measures. We hypothesized that there would be moderate to strong correlations between scores for the WALS and the three work measures and moderate correlations with perceived health status and condition-specific symptoms and physical function scales. Correlations of 0.4–0.59 are considered moderate and ≥0.6 are strong [41].

Discriminant validity (i.e. hypothesis testing that there would be significant WALS score differences between those reporting they had very poor/poor, fair or good/very good perceived health status). This was assessed using Kruskal–Wallis tests, with *P* ≤ 0.05 considered significant.

#### Reliability

Internal consistency (i.e. the degree of interrelatedness between items within a scale) was assessed using Cronbach’s α. Results ≥0.8 were deemed good to excellent: ≥0.9 is consistent with individual use; and >0.7 with group-level use [41].

Test–retest reliability is the extent to which scores for participants who report that their health has not changed are the same for repeated measurements over time. This was assessed in those reporting perceived health as ‘the same’ at T2, using Spearman’s correlations and intraclass correlation coefficient (ICC) (2,1): two-way random consistency, average measures model. An ICC of ≥0.75 is considered excellent and 0.5–0.74 moderate [42]. Reliability of individual WALS items was calculated using linear weighted κ, with levels of agreement considered as: 0.41–0.60 = moderate; ≥0.61 = good [41].

#### Responsiveness

Sensitivity to change was assessed by calculating the standard error of measurement (SEM) and the minimal detectable change_95_ (MDC_95_) scores. The SEM represents the s.d. of repeated measures of one individual. The MDC_95_ is a statistical estimate of the smallest detectable change corresponding to change in ability rather than a measurement error [43, 44].

Floor and ceiling effects were considered present if >15% of participants achieved either the lowest or highest scores in the WALS [45]. If present, these can have a negative effect on the quality of the measure, because responsiveness (i.e. the ability to detect change over time) will be limited.

## Results

### Phase 1

Linguistic validation, cross-cultural adaptation and content validity results are given in Supplementary Data S1, Tables S2 and S3, available at *Rheumatology Advances in Practice* online. In cognitive debriefing interviews (*n* = 48; participant characteristics are in Table 1), all items were considered very relevant and, following expert panel review, only minor changes in wording were needed.

**Table 1.** Phase 1 and 2 participant demographic data

Parameter	RA		axSpA		OA		FM	
	Phase 1	Phase 2	Phase 1	Phase 2	Phase 1	Phase 2	Phase 1	Phase 2
*n* =	12	294	10	199	13	173	13	156
Sex, *n* (%), male:female	5:7	76 (26.00):218 (74.00)	4:6	124 (62.30): 75 (37.70)	4:9	54 (31.00):119 (69.00)	2:11	10 (6.00): 146 (94.00)
Age, mean (s.d.), years	57.33 (6.77)	53.47 (8.97)	33.00 (14.62)	46.96 (10.24)	55.92 (6.70)	56.49 (7.21)	39.69 (9.11)	45.71 (10.05)
Job skill level, *n* (%)								
1 and 2	3	149 (51.00)	5	66 (33.10)	8	84 (48.60)	7	95 (61.00)
3 and 4	9	142 (48.00)	5	133 (66.90)	5	88 (50.80)	6	61 (39.00)
Missing	–	3 (1.00)	–	–	–	1 (0.70)	–	–
Disease duration, mean (s.d.), years	18.08 (11.93)	7.66 (7.97)	12.70 (9.78)	12.33 (10.40)	12.35 (10.60)	4.97 (6.83)	5.38 (3.55)	2.99 (4.17)
Phase 2 only								
Symptom duration, mean (s.d.), years	9.33 (8.52)		18.97 (11.75)		7.89 (8.50)		8.36 (7.16)	
Living conditions, *n* (%)								
With spouse/family/significant other	241 (82.00)		179 (89.90)		143 (83.00)		139 (89.00)	
Children <18 years old living at home, *n* (%)	69 (23.00)		68 (34.20)		31 (18.00)		56 (36.00)	
Educational level, *n* (%), ISCED								
No formal qualifications	27 (9.20)		14 (7.00)		17 (10.00)		7 (4.00)	
Secondary/post-secondary non-tertiary	148 (50.30)		100 (50.30)		91 (53.00)		76 (49.00)	
Tertiary	117 (39.80)		84 (42.20)		61 (35.00)		73 (47.00)	
Missing	2 (0.70)		1 (0.50)		4 (2.00)		–	
Full-time:part-time work, *n* (%)	160 (54.40):134 (45.60)		150 (75.40):49 (24.60)		106 (61.30):67 (38.70)		70 (45.00):86 (55.00)	
Hours worked, mean (s.d.)	33.24 (12.47)		37.77 (10.44)		34.16 (11.66)		31.50 (10.56)	
Self-employed, *n* (%)	63 (21.40)		34 (17.10)		21 (12.10)		18 (11.50)	
Physical demands of job, *n* (%)								
None/a little	101 (34.40)		83 (41.70)		53 (30.70)		61 (39.10)	
Noticeable	37 (12.60)		175 (8.90)		22 (12.70)		14 (9.00)	
A lot/great deal	156 (53.00)		99 (49.80)		98 (56.60)		81 (51.90)	
Medication regimen, *n* (%)								
None	2 (0.70)		19 (9.50)		33 (19.00)		23 (15.00)	
NSAIDs ± analgesics	11 (3.70)		4 (2.00)		118 (69.00)		14 (9.00)	
CSs ± NSAIDs	6 (2.00)		51 (25.60)		10 (6.00)		6 (4.00)	
Single DMARD	103 (35.00)		10 (5.00)		–		–	
Combination DMARD	97 (33.00)		2 (1.00)		–		–	
Biologic/biosimilar	66 (22.40)		112 (56.30)		–		–	
Neuropathic analgesics (e.g. gabapentin/pregabalin)	–		–		12 (7.00)		99 (64.00)	
FM, opiate medication	–		–		–		12 (8.00)	

axSpA: axial spondyloarthritis; ISCED: International Standard Classification of Education.

### Phase 2

Overall, 1359 people were referred to the study; 831 returned T1 and 622 T2 questionnaire booklets (Supplementary Fig. S1, available at *Rheumatology Advances in Practice* online). The response rates were as follows: secondary care, 62% (696/1117); community hospitals, 53% (119/224); and volunteers, 89% (16/18). Participant characteristics are shown in Table 1 and work and health measures in Table 2. The median time between tests was 36 (IQR 28–47) days.

**Table 2.** Phase 2: participants’ work and health measures

Parameter	RA (*n* = 294)	axSpA (*n* = 199)	OA (*n* = 173)	FM (*n* = 156)
**Work measures**				
WALS, 0–36, median (IQR)	9.00 (5.00–14.00)	6.00 (3.00–11.00)	10.00 (6.00–14.00)	16.00 (12.00–19.00)
WLQ-25, 0–100, median (IQR)				
Time management demands	30.00 (10.00–55.00)	25.00 (5.00–50.00)	30.00 (10.00–50.00)	60.00 (40.00–80.00)
Physical demands	37.50 (20.00–58.33)	37.50 (12.50–55.31)	41.67 (25.00–58.33)	58.33 (43.75–73.96)
Mental interpersonal demands	16.67 (5.55–36.11)	13.89 (2.78–30.56)	16.67 (5.56–36.11)	44.44 (27.78–61.11)
Output demands	20.00 (5.00–44.06)	10.00 (0–30.00)	20.00 (5.00–43.75)	45.00 (25.00–65.00)
WLQ-25 percentage productivity loss	6.92 (3.27–11.12)	5.40 (1.71–9.36)	6.65 (3.43–11.40)	13.26 (9.20–16.53)
WLQ-25 summed score	29.38 (14.17–43.70)	22.74 (7.08–40.03)	28.61 (15.21–45.36)	51.69 (37.30–64.62)
WIS (0–23 RA, OA, FM; 0–20 AS), median (IQR)	13.00 (7.75–18.00)	11.00 (4.00–15.00)	13.00 (8.00–17.00)	18.00 (15.00–20.00)
Low work instability, *n* (%)	95 (32.30)	99 (49.70)	59 (34.10)	6 (3.84)
Moderate work instability, *n* (%)	123 (41.80)	80 (40.20)	79 (45.70)	64 (41.00)
High work instability, *n* (%)	76 (25.90)	20 (10.10)	35 (20.20)	86 (55.16)
WPAI, median (IQR)				
Percentage overall work impairment owing to health	30.00 (10.00–60.00)	20.00 (0–40.00)	30.00 (10.00–58.11)	66.15 (50.00–80.00)
**Health measures**				
Perceived severity health last month (1–5), median (IQR), *n* (%)	3.00 (2.00–3.00)	2.00 (2.00–3.00)	3.00 (3.00–3.00)	4.00 (3.00–4.00)
Poor/very poor	45 (15.30)	21 (10.60)	37 (21.40)	83 (53.00)
Fair	133 (45.20)	78 (39.20)	95 (54.90)	63 (41.00)
Good/very good	116 (39.50)	100 (50.30)	41 (23.70)	10 (6.00)
RA				
RAID (0–10), median (IQR)	4.84 (3.15–6.42)	–	–	–
HAQ20 (0–60), median (IQR)	9.00 (3.00–18.00)	–	–	–
axSpA				
BASDAI (0–10), median (IQR)	–	3.93 (1.95–5.87)	–	–
BASFI (0–10), median (IQR)	–	2.97 (1.40–5.35)	–	–
OA				
WOMAC, median (IQR)				
Pain (0–20)	–	–	10.00 (7.00–13.00)	–
Physical function (0–68)	–	–	31.00 (21.00–41.50)	–
FM				
FIQR, normalized scores, median (IQR)				
Overall impact (0–20)	–	–	–	14.00 (10.00–17.00)
Symptoms (0–50)	–	–	–	34.50 (28.13–39.00)
Function (0–30)	–	–	–	19.33 (14.67–22.67)
FIQR total (0–100)	–	–	–	68.33 (54.20–77.50)
T1 to T2	*n* = 219	*n* = 156	*n* = 131	*n* = 116
Time between T1 and T2, median (IQR), days	40.00 (34.00–48.00)	38.00 (29.00–49.25)	30.00 (23.75–37.00)	33.00 (26.50–45.00)
Perceived change in health status at T2 *vs* T1, *n* (%)				
Much/somewhat less troublesome	36 (16.44)	24 (15.39)	16 (12.10)	14 (12.07)
The same^a^	136 (62.10)	99 (63.47)	78 (59.10)	54 (46.55)
Somewhat/much more troublesome	47 (21.46)	31 (19.88)	38 (28.80)	48 (41.38)
Missing	–	2 (1.00)	–	–

For all measures, higher scores indicate more work/health problems; WIS (RA-WIS used for RA, AS and FM; AS-WIS for AS). WIS cut-points: low instability: RA-WIS <10; AS-WIS <11; moderate instability: RA-WIS 10–17; AS-WIS 11–18; high instability: RA-WIS >17; AS-WIS = 19–20.

a Participants included in test–retest reliability analysis.

axSpA: axial spondyloarthritis; FIQR: Fibromyalgia Impact Questionnaire—Revised; IQR: interquartile range; NRS: numerical rating scale; RAID: RA Impact of Disease; WALS: Workplace Activity Limitations Scale; WIS: Work Instability Scale; WLQ-25: Work Limitations Questionnaire-25; WPAI: Work Productivity Activity Impairment.

#### Compliance

There were 0.01% missing data. WALS scores could not be calculated for three participants (with 5–12 missing items each) in each of the RA, axSpA and OA groups. These participants were not included in analyses (i.e. the sample size was reduced to 822). All FM scores could be calculated. The frequency of ‘not applicable’ (re-scored as 0) and ‘missing’ data are shown in Supplementary Table S4*,* available at *Rheumatology Advances in Practice* online.

### Validity

#### Construct (structural) validity

Table 3 displays the detailed analysis of fit to the Rasch model. The scale is unidimensional. The items most easily affirmed (i.e. the transition from no to some difficulty) were: ‘Lifting, carrying or moving objects’ (RA); ‘Crouching, bending or kneeling’ (axSpA and OA); and ‘Concentrating’ (FM). The items most difficult to affirm (i.e. the transition from a lot of difficulty to unable to do) was: ‘Working with your hands’ (RA, axSpA, OA and FM). Invariance was confirmed for age, sex, condition, disease duration, educational and work status. Full details of the results are given in Supplementary Data S3, available at *Rheumatology Advances in Practice* online. A transformation table was created to convert WALS raw scores to interval level scores, if required (Supplementary Table S5, available at *Rheumatology Advances in Practice* online). A reference metric was also created to allow test equating of raw WALS scores with raw RA- and AS-WIS scores (Supplementary Table S6, available at *Rheumatology Advances in Practice* online). Both the latter have clinically derived cut-points. Direct comparison with these cut-points suggests that WALS scores of 7 and 14 would indicate thresholds for moderate and high work instability, respectively, in these four RMDs.

**Table 3.** Fit of the Workplace Activity Limitations Scale to the Rasch model: construct (structural) validity

Diagnosis	Residuals (s.d.)		χ^2^		Reliability		Dimensionality	DIF	ECV	Latent correlation^a^
	Item	Person	Value (d.f.)	*P*-value	PSI	α	% *t*-tests (LCI)			
RA	0.01	0.84	28.10 (20)	0.11	0.83	0.87	2.70	None	0.97	0.93
axSpA	0.43	0.73	18.70 (15)	0.24	0.77	0.85	3.52	None	0.93	0.90
OA	0.17	0.92	22.80 (19)	0.24	0.80	0.83	1.80	None	0.96	0.92
FM	0.19	0.81	13.40 (18)	0.77	0.80	0.80	3.21	None	0.98	0.95
Across all four conditions	0.41	0.97	24.10 (23)	0.40	0.84	0.87	2.80	None	0.97	0.95
Ideal values	**1.0**	**1.0**	–	**>0.05**	**>0.7**	**>0.7**	**<0.5**	–	**>0.9**	**>0.9**

Bold text indicates ideal values.

a Between parallel forms.

α: Cronbach’s α; axSpA: axial spondyloarthritis; DIF: differential item functioning; ECV: explained common variance; LCI: lower confidence interval; PSI: Person separation index.

#### Concurrent validity

As hypothesized, the WALS exhibited a moderate to strong positive correlation with work measures (total scores: *r*_s_ = 0.51–0.84), perceived health status (*r*_s_ = 0.42–0.71) and diagnosis-specific symptoms (*r*_s_ = 0.54–0.68) and physical function measures (*r*_s_ = 0.55–0.77) (Table 4).

**Table 4.** Concurrent validity of the Workplace Activity Limitations Scale with work and health measures

WALS (0–36) correlations with:	RA (*n* = 294)	axSpA (*n* = 199)	OA (*n* = 173)	FM (*n* = 156)
	*r*_s_	*r*_s_	*r*_s_	*r*_s_
**Work measures**				
WLQ-25 (0–100)				
Time management demands	0.70**	0.75**	0.65**	0.57**
Physical demands	0.62**	0.73**	0.50**	0.39**
Mental interpersonal demands	0.68**	0.71**	0.62**	0.58**
Output demands	0.71**	0.71**	0.52**	0.56**
WLQ-25 percentage productivity loss	0.78**	0.83**	0.63**	0.64**
WLQ-25 summed score	0.79**	0.84**	0.67**	0.66**
WIS (0–23 RA, OA, FM; 0–20 axSpA)	0.77**	0.84**	0.73**	0.60**
WPAI (%)				
Overall work impairment owing to health	0.65**	0.80**	0.68**	0.51**
**Health measures**				
Self-reported health in last month (1–5)	0.61**	0.71**	0.53**	0.42**
RA				
RAID (0–10), median (IQR)	0.68**	–	–	–
HAQ20 (0–60), median (IQR)	0.73**	–	–	–
axSpA				
BASDAI (0–10), mean (s.d.)	–	0.68**	–	–
BASFI (0–10), mean (s.d.)	–	0.77**	–	–
OA				
WOMAC, median (IQR)				
Pain (0–20)	–	–	0.56**	–
Physical function (0–68)	–	–	0.55**	–
FM				
FIQR, normalized scores				
Overall impact (0–20)	–	–	–	0.43**
Symptoms (0–50)	–	–	–	0.54**
Function (0–30)	–	–	–	0.55**
FIQR total (0–100)	–	–	–	0.61**

** Correlation significant at 0.01 level.

axSpA: axial spondyloarthritis; FIQR: Fibromyalgia Impact Questionnaire—Revised; RAID: RA Impact of Disease; *r*_s_: Spearman’s correlations; WALS: Workplace Activity Limitations Scale; WIS: Work Instability Scale; WLQ-25 = Work Limitations Questionnaire-25; WPAI: Work Productivity Activity Impairment.

#### Discriminant validity

As hypothesized, there were significant differences between the three levels of perceived disease severity for the WALS across all four conditions, with higher perceived disease severity subgroups scoring worse (Supplementary Table S7, available at *Rheumatology Advances in Practice* online).

### Reliability

#### Internal consistency

Cronbach’s α values across the four conditions were good to excellent, ranging from 0.80 (FM) to 0.87 (RA). All were consistent with group-level use (Table 3).

#### Test–retest reliability

At T2, 356 of 622 (57%) participants reported that their condition was ‘the same’ as at T1 and included in analyses. For all four conditions, correlations between T1 and T2 scores were strong to very strong (*r*_s_ = 0.80 and above). The ICCs (2,1) were excellent, at 0.90 and above (Table 5). Item reliability was moderate to good (Supplementary Table S8, available at *Rheumatology Advances in Practice* online).

**Table 5.** Test–retest reliability and sensitivity to change of the Workplace Activity Limitations Scale

Condition	Number for test–retest^a^	Test 1 score^a^ [median (IQR)]	Test 2 score^a^ (median, IQR)	Spearman’s correlation^a^, *r*_s_	ICC (2,1)^a^ (95% CI)	SEM^a^	MDC_95_
RA	136	8.00 (4.00–12.75)	7.00 (4.00–11.00)	0.83**	0.92 (0.90, 0.94)	1.15	3.17
axSpA	98	5.00 (2.00–9.00)	5.00 (1.75–7.25)	0.80**	0.90 (0.84, 0.93)	1.67	4.82
OA	78	8.00 (5.75–12.25)	7.00 (4.00–12.25)	0.81**	0.90 (0.84, 0.93)	1.83	5.08
FM	54	15.00 (10.00–19.25)	14.00 (11.00–19.00)	0.82**	0.90 (0.83, 0.94)	1.57	4.36

a Participants indicating perceived health ‘about the same’ at T1 and T2, who had WALS scores available at both time points.

** Correlation significant at 0.01 level.

axSpA: axial spondyloarthritis; ICC: intraclass correlation coefficient; IQR: interquartile range; MDC_**95**_: minimum detectable change; SEM: standard error of measurement.

### Responsiveness

#### Sensitivity to change

The MDC_95_ scores ranged from 3.17 (RA) to 5.08 (OA) in those stating that their health was ‘the same’ at T2 (Table 5).

#### Floor and ceiling effects

Fewer than 15% scored 0 for the WALS, indicating there was no floor effect: RA = 6 of 294 (2%); axSpA = 21 of 199 (10.40%); OA = 3 of 176 (1.70%); and FM = 1 of 156 (0.60%). There were no ceiling effects (i.e. score of 36) in the four conditions.

## Discussion

A linguistically validated British English version of the WALS is now freely available for use in the UK (Supplementary Data S2, available at *Rheumatology Advances in Practice* online and www.mskhub.com). This study provides new evidence that the British English WALS has good psychometric properties in RA, OA, axSpA and FM and can be used in the UK.

We ensured linguistic and cross-cultural validity of the WALS by using a standard translation process [21], with the approval of the WALS developer. Example activities were updated in three items: to reflect active travel options (in item 1); and in items 2 and 6 to increase relevancy to manual jobs. Participants considered the WALS comprehensive, comprehensible and easy to complete, indicating good content validity from the perspective of the patients in these four RMDs (i.e. comparable to findings in RA and OA in Canada [9]).

To our knowledge, this is the first study examining construct (structural) validity of the WALS in RA, axSpA, OA and FM, demonstrating fit to the Rasch model and making available a Rasch transformation table from WALS raw to interval scores. Given that the WALS is unidimensional, either summed or (Rasch) standardized scores can be used. As hypothesized, the WALS demonstrated good concurrent validity with other work measures, except the WLQ-25 physical demands subscale in FM. Some participants can have difficulty completing this subscale, because instructions are reversed compared with the other three subscales [6]. Potentially, more participants with FM experience such difficulty, because >50% of people with FM report cognitive deficits, which is higher than that experienced by people with RA, for example [46, 47]. As hypothesized, correlations with physical function, symptom and health scales were moderate in OA and FM, but generally strong (i.e. higher than expected) in RA and axSpA. These findings are comparable to those in RA and OA in Canada [17]. We also demonstrated the WALS has good discriminant validity in the four RMDs, which had not been tested previously.

Internal consistency was good, and comparable to findings in RA and OA in Canada [6], meaning that the WALS can be used for group measurement in the four RMDs. Identifying that WALS scores of 7 and 14 equate to RA- and AS-WIS cut*-*points for moderate and high work instability indicates that the WALS could help not only to identify patients’ work limitations but also who could benefit from work rehabilitation. The evidence for test–retest reliability is extended, and specific values of MDC_95_ for each of the four RMDs are provided. These had previously been tested in only a small sample of ‘workers with arthritis’ [5, 6].

It is worth noting that across all four RMDs, the phase 2 results showed that those reporting they had ‘fair’ health status had average WALS scores exceeding the cut-off score for moderate work instability (i.e. 7) and also (apart from axSpA) those with poor*/*very poor health status had scores exceeding the cut-off for high work instability (i.e. 14). The FM group also had higher average WALS scores than the other three RMDs, despite being younger, with many experiencing high work instability. Health professionals working with employed people with RMDs reporting fair or poor health status, and especially those with FM, are recommended to screen for work problems and provide work advice and support, as relevant.

The WALS tests intrinsic work activity impairment (i.e. capacity in International Classification of Functioning, Disability and Health terms), because the instructions specify reporting difficulty without help from another person or use of gadgets or equipment. It might not therefore reflect the person’s real ability to work (performance in International Classification of Functioning, Disability and Health terms; i.e. with ergonomic modifications, help and*/*or job accommodations). Under the UK Equality Act 2010, it is the duty of an employer to provide these (termed as ‘reasonable adjustments’) to employees with disabilities. Clinically, and in work rehabilitation studies, using a WALS omitting the instructions to answer ‘without help or gadgets*/*equipment’ could better identify whether improvement occurs following work rehabilitation and putting reasonable adjustments in place. Modified instructions could focus on how people usually do these activities. Additionally, there is no time frame in the instructions. Some work measures (e.g. the WLQ-25), ask about the last 2 weeks. A disadvantage of a short time frame is, firstly, that the measure can be completed only by people working for ≥1 day during that time. Those on sick, annual or other extended leave for >2 weeks cannot complete it. Second, people with RMDs can experience episodic flares or worse health. A limited time frame means that completion might coincide with a period of unusual ill-health or good health. Avoiding a time scale overcomes this problem, because participants might either reflect on their difficulties when last at work or estimate difficulties. This could, however, be problematic in those on long-term sick leave if they estimate difficulties incorrectly. Particularly in intervention studies, it might be better to specify a time (e.g. 3 months). Future research could psychometrically test a WALS with modified instructions.

A strength of this study was that we had relatively large samples of people with RA, axSpA, OA and FM recruited from a wide variety of NHS outpatient clinics, meaning that the results are representative for people accessing secondary or community care. Limitations were that fewer people with FM had stable self-reported health between T1 and T2, compared with the other conditions, resulting in a smaller sample for test–retest reliability than required. Responsiveness (i.e. longitudinal validity) still needs testing, and minimal clinically important differences need to be established in the UK. Further testing in other RMDs is required.

### Conclusions

Overall, psychometric testing of the British English WALS demonstrated good validity and reliability in employed people with RA, axSpA, OA and FM in the UK. The WALS meets most recommendations of the Consensus-based Standards for the selection of health Measurement Instruments (COSMIN) checklists for methodological quality and reporting [19, 20]. Accordingly, the British English WALS can be used in the UK for these four RMDs.

## Supplementary Material

rkad028_Supplementary_Data

## Data Availability

The datasets generated during and/or analysed during the current study are available from the corresponding author on reasonable request, following completion of associated studies. The British English WALS is available in the Supplementary Materials.
