# Supplementary material for: Psychometric testing of the British English Workplace Activity Limitations Scale in four rheumatic and musculoskeletal conditions
Source: Rheumatol Adv Pract. 2023 Mar 10;7(1):rkad028. doi: 10.1093/rap/rkad028 (PMC10027431; doi:10.1093/rap/rkad028)
Supplement: rkad028_Supplementary_Data [file rkad028_supplementary_data.docx]

**Supplementary Materials:**

Psychometric testing of the British-English Workplace Activity Limitations Scale in four rheumatic and musculoskeletal conditions. Hammond A, Tennant A, Ching A, Parker J, Prior Y, Gignac M, Verstappen S, O’Brien R. Rheumatology Advances in Practice. 2023.

**Contents**

| **Page:** |  |
| --- | --- |
| **2** | Supplementary File S1: Linguistic validation, cross cultural adaptation, and content validity |
| **5** | Supplementary File S2: Workplace Activity Limitations Scale (British English). |
| **7** | Supplementary File S3: Rasch Analysis methods and results |
| **11** | Supplementary Table S1: The hierarchical analytical structure for achieving ft of the Workplace Activity Limitations Scale to the Rasch model |
| **12** | Supplementary Figure S1: Recruitment Flowchart Phase 2 |
| **13** | Supplementary Table S2: Phase 1 participants’ responses regarding relevance of Workplace Activity Limitations Scale items (n = 48) |
| **14** | Supplementary Table S3: Content validity of Workplace Activity Limitations Scale linked to the ICF Core Set |
| **17** | Supplementary Table S4: Frequency of “not applicable” and missing items in the Workplace Activity Limitations Scale |
| **18** | Supplementary Table S5. Raw score to interval scale transformation of the Workplace Activity Limitations Survey. |
| **19** | Supplementary Table S6: Calibration of the Workplace Activity Limitations Scale and Work Instability Scales on the Reference Metric. |
| **20** | Supplementary Table S7: Discriminant validity of the Workplace Activity Limitations Scale |
| **21** | Supplementary Table S8: Workplace Activity Limitations Scale item test-retest reliability (quadratic weighted kappa). |

**Supplementary File S1: Linguistic validation, cross cultural adaptation, and content validity.**

**Method: Linguistic validation, cross-cultural adaptation, and content validity**

The following procedures were used [1]:

*Forward translation*: two translators (a rheumatology researcher familiar with the WALS (AH), and a non-health professional (experienced teacher, including of English: JG) unfamiliar with the WALS) independently reviewed the WALS to identify words requiring changing into British English and use of Plain English (i.e., simplifying words and phrases).

*Translation synthesis*: the two translators discussed and agreed recommended changes.

*Backward translation*: was not required as the translation was into another form of English.

*Expert committee review*: The committee included: one translator (AH); three occupational therapists experienced in work and musculoskeletal conditions (YH, TW, RO’B); the WALS developer MG: Canadian-English speaker); experienced PROMS researchers (AT, AH, YP, SV) and two patient research partners (AP, SK). The committee discussed the synthesised translation, made additional recommendations, and agreed and approved the draft British English WALS. This process ensures semantic, idiomatic, experiential, and conceptual equivalence.

*Field testing of the draft WALS and content validity*: Cognitive debriefing interviews were used to investigate the WALS from people with RMD’ perspectives [2]. PROM content validity should be assessed by experts, i.e., patient/ public representatives of the target populations [3]. At least 10 in each target group should be included [4]. Participants were mailed a paper questionnaire booklet, including the draft British English WALS, to complete at home, and asked to consider WALS ease of completion, item relevance and if anything important was missing. Within two weeks, they were interviewed, face-to-face or by telephone, about comprehensiveness (1 = not relevant; 5 = extremely relevant; and any missing items) and comprehensibility (instructions, content, layout). Findings were discussed with the expert committee, further changes made and the final British English WALS agreed.

Content validity was further examined by linking the WALS to the Activities and Participation component of the International Classification of Functioning, Disability and Health (ICF) Core Set for Vocational Rehabilitation [5,6]. The Flesch-Kincaid Grade score was calculated using Microsoft Word to check readability was similar to the original WALS [7].

**Results**

Following forward translations and synthesis, the expert panel reviewed these and agreed the following changes: to have a root question “how much difficulty do you have…”, rather than each item starting with this; item 1 (travel) changing “subway” to “train” and including active travel (walking, cycling); item 2 (get around the workplace) changing “hallways” to “corridors” and adding “machinery” to include physical work settings; item 6 (work with hands) widening examples to include keyboard/touchscreen (as most jobs now involve computer usage), tools and operating machinery (to be inclusive of physical work) and adding smartphone to “hold a phone”; item 9 (managing hours of work) changing “schedule” to ”shifts,” as more commonly used in British English; and item 12 (concentration) changing “due to arthritis” to “condition”, as those with FM may not consider their diagnosis as arthritis.

Cognitive debriefing interviews were conducted with 48 participants (face-to-face n = 6; telephone n = 42) (Table 1), with results reviewed by the expert panel to determine any further changes in the WALS required. Most participants considered the WALS comprehensive, with items very or extremely relevant for their condition, with no significant differences between groups (Supplementary Table S2). Only six suggested additional items, although only by one each and so not included. These were: “driving for work”; “parking near work”; “opening door handles, bottles and jars”; “going to the [work] bathroom (turning door handles and taps)”; “dealing with co-workers and the public” and “having to explain myself [condition] to people.” Most (43/48) considered the WALS comprehensible, with instructions, content, and layout easy to understand. Only five stated these were “partly easy.” Of these, one recommended moving the “not applicable” column to the first response option, rather than last, which was changed. As only one person each raised the following issues, no changes were made: one participant (axSpA) misunderstood instructions, indicating all activities were “not applicable” as able to do them despite pain; and another participant (FM) reported taking several attempts to assimilate longer items (e.g., item 6). Three noted problems with the response options, with one each stating: the gap between “some” and “a lot” is too large and an intermediate option needed; frequency of difficulty rather than amount would be better; and preferring a focus on ability rather than difficulty. No problems were reported with the lack of time frame in the instructions.

The WALS was linked to 16 items in the ICF Core Set for Vocational Rehabilitation, indicating reasonable coverage, although it could potentially be linked to a further 14 items dependent on how a person interprets item 11 (managing job demands) in relation to their job (Supplementary Table S3). The Flesch-Kincaid Grade Level score was 7.6, similar to the original WALS at 7.1, indicating a reading age of 11- to 13-year-olds [7].

**References**

1. Beaton DE Bombardier C, Guillemin F, Ferraz MB. Recommendations for the Cross-Cultural Adaptation of the DASH & QuickDASH Outcome Measures. 2007. Toronto, ON: Institute of Work and Health. <https://dash.iwh.on.ca/sites/dash/files/downloads/cross_cultural_adaptation_2007.pdf>

Accessed 6 September 2022.

1. Acquadro C, Joyce CRB, Patrick DL, Ware JE, Wu AW. **Linguistic Validation Manual for Patient-Reported Outcomes (PRO) Instruments.** Mapi Research Trust, Lyon, France. 2004.
2. De Vet HCW, Terwee CB, Mokkink LB, Knol DL, Measurement in Medicine: a practical guide. Cambridge (UK): Cambridge University Press; 2011
3. Terwee CB, Mokkink LB, Knol DL, et al. Rating the methodological quality in systematic reviews of studies on measurement properties: a scoring system for the COSMIN checklist. *Qual Life Res* 2012;21:651-657.
4. Finger ME, Escorpizo R, Glässel A, Gmünder HP, Lückenkemper M, Chan C, Fritz J, Studer U, Ekholm J, Kostanjsek N, Stucki G, Cieza A. ICF Core Set for vocational rehabilitation: results of an international consensus conference, Disabil Rehabil 2012:34; 429-438
5. **Cieza A, Geyh S, Chatterji S, Kostanjsek N, Ustun B, Stucki G. ICF linking rules: an update based on lessons learnt. J Rehabil Med 2004;37(4):212-218**
6. JISC Tech Dis Inclusion Technology Advice. How to use the Readability statistics. 2013. <https://sshs.exeter.ac.uk/media/universityofexeter/schoolofsportandhealthsciences/documents/Guidelines_for_checking_readability_of_Participant_Information_Sheets.pdf>. Accessed 6 September 2022.

**Supplementary File S2: Workplace Activity Limitations Scale - British English.**

These questions ask you about activities related to your job. When you think about how much difficulty you have with these activities, think about doing them **WITHOUT** any help from another person or **WITHOUT** the help of a special gadget or piece of equipment.

***Please tick: the most appropriate box; and tick if any difficulty is due to your musculoskeletal condition/ arthritis.***

***Tick*** *“Not applicable” only if the question describes something* ***not*** *part of your work.*

| ***How much difficulty do you have:*** | ***Not applicable*** | ***No difficulty*** | ***Some difficulty*** | ***A lot of difficulty*** | ***Unable to do*** | ***Difficulty unrelated to musculoskeletal condition/ arthritis*** |
| --- | --- | --- | --- | --- | --- | --- |
| 1. Getting to and from work (e.g., train, bus, car, cycle, walk) and getting to and from work on time? |  |  |  |  |  | Yes No |
| 2. Getting around the workplace (e.g., stairs, corridors, furniture, machinery)? |  |  |  |  |  | Yes No |
| 3. Sitting for long periods of time at your job (e.g., more than 20 minutes)? |  |  |  |  |  | Yes No |
| 4. Standing for long periods of time at your job (e.g., more than 20 minutes)? |  |  |  |  |  | Yes No |
| 5. Lifting, carrying, or moving objects? |  |  |  |  |  | Yes No |

| ***How much difficulty do you have:*** | ***Not applicable*** | ***No difficulty*** | ***Some difficulty*** | ***A lot of difficulty*** | ***Unable to do*** | ***Difficulty unrelated to musculoskeletal condition/ arthritis*** |
| --- | --- | --- | --- | --- | --- | --- |
| 6. Working with your hands (e.g., writing, using a keyboard/ touchscreen, grasping small objects/ tools, operating machinery, holding a phone/ smartphone)? |  |  |  |  |  | Yes No |
| 7. Crouching, bending, kneeling or  working in awkward positions? |  |  |  |  |  | Yes No |
| 8. Reaching? |  |  |  |  |  | Yes No |
| 9. Managing the shifts or hours of work your job requires? |  |  |  |  |  | Yes No |
| 10. Managing the pace of work your job requires? |  |  |  |  |  | Yes No |
| 11. Meeting your current job demands? |  |  |  |  |  | Yes No |
| 12. Concentrating or keeping your mind on your work (because of your condition)? |  |  |  |  |  | Yes No |

**Scoring instructions:**

Items are scored: no difficulty = 0; some difficulty = 1; much difficulty = 2; unable to do = 3. Items are summed to form a 0-36 scale. High scores indicate greater work limitations. “Not applicable” items are scored as 0, as the activity does not therefore present difficulty for the person. “Difficulty unrelated to condition/ arthritis” is for information **only**, as the person is still experiencing work difficulties, whatever the cause. The WALS is considered a measure of presenteeism.

A Rasch transformation table is available to convert WALS raw scores to interval scores (Hammond et al, 2023).

A score of 0-6 indicates low work stability; 7 – 13 moderate work instability: and 14-36 high work instability.

**Missing data:** up to three items are allowed. Missing items are replaced by either the person’s overall median or mean WALS score, dependent on analysis approach.

**© Hammond A, Tennant A, Prior Y, Gignac M 2023.**

**Supplementary File S3: Workplace Activity Limitations Scale: Rasch Analysis methods and results.**

**Method**

Data was tested against the requirements of the Rasch Measurement model [1]. Briefly, these requirements include i) unidimensionality; ii) monotonicity; iii) homogeneity; iv) local independence and v) group invariance [2, 3]. Items added together to provide a score should satisfy all of these requirements. That is, they should i) measure one thing (domain/construct/trait; ii) the probability of a positive response to an item (or in the case of polytomous (i.e., two or more ordinal categories) items, the transition from one response category to the next) should increase with underlying ability, as should the total score [4], iii) the same hierarchical ordering of items should hold for each level (or grouping) of the score [5]; iv) items should be conditionally (on the score) independent of one another [6] and v) the response to items across different groups such as age or gender should, conditioned on the total score, be the same – referred to as (the absence of) Differential Item Functioning (DIF) [3].

Each requirement is tested. A t-test is used to determine if two separate groups of items deliver significantly different estimates, following the procedure given by Smith [7]. The hierarchical ordering of items across the scale is determined through a Chi-Square test of fit based on grouped scores. Monotonicity is evaluated through inspection of the item-category ordering. Conditional item dependence is determined though the correlation of residuals, where pair-wise correlations should not exceed 0.2 above the average residual [8]. Should clusters of locally dependent items be found, consideration is given to grouping these into ‘super items’ or testlets (simply adding them together to make one larger item, the latter based on a priori defined groups) to absorb the local dependency [9]. In the RUMM2030 software, this gives a bi-factor equivalent solution retaining a specified proportion of the variance. This “Explained Common Variance (ECV)” is reported, whereby a value less than 0.7 is indicative of requiring a multidimensional model, a value above 0.9 a unidimensional model, and the grey area in between, undetermined, requiring further evidence [10]**.** Consequently, a value of the ECV at 0.9 and above is considered acceptable in the current analysis. Where possible when two parallel forms are created from the pattern of local dependency in the item set, requires a latent correlation ≥ 0.9. This is consistent with the reliability required for individual use [11-14]. Consequently, valid parallel forms would require both their latent correlation to be ≥0.9 and the ECV to be ≥0.9.

Group invariance (DIF) is tested through an analysis of variance (ANOVA) of residuals for age, gender, duration, education- and job skill- levels, and whether or not the patient is self-employed or employed, and full-time and part-time. Should DIF be identified it is tested by a comparison of person estimates from split and unsplit solutions to see if it is ‘substantive’ [14,15]. Where the difference is significant (a paired t-test), the result is reported as an effect size where a value higher than 0.1 is considered to represent substantive DIF [16]. If this is present, then the scale works in different ways for the contextual factor under consideration, and results are reported separately.

Given the requirements for fit, a hierarchical strategy was used to achieve fit to the model (**Supplementary Table S1**). With level 1 as the priority, all requirements listed above for fit to the model must be met. Should a Level 5 solution be unavailable, item deletion will be considered (Level 6). If this fails, then Level 7 will be utilised to test if the scale satisfies ordinal scaling; and if this fails then Level 8 indicates no valid ordinal scale. Data were fitted for the WALS scale within each condition.

**Results**

The initial fit of the WALS to the Rasch model showed multidimensionality, caused by clusters of locally independent items in both the upper and lower part of the scale. Consequently, fit of the WALS items to the Rasch model in those with RA, axSpA, OA and FM was at level 4 (i.e., local-dependency cluster based-parallel form: Table 3; Supplementary Table S1). The items most easily affirmed (i.e., the transition from no to some difficulty) were: ‘Lifting, carrying, or moving objects’ (RA); “Crouching, bending or kneeling” (axSpA, OA); and “Concentrating” (FM). The items most difficult to affirm (i.e., the transition from a lot of difficulty to unable to do) was: “Working with your hands” (RA, axSpA, OA and FM), particularly in FM, as the transition was five logits higher than the next threshold. No invariance (DIF) was observed in any condition. Local item dependency was observed necessitating the grouping of items into “super items.” The dependencies were observed in the lower and upper part of the scale. For example, the items ‘Managing the pace of work that your job requires’ and ‘Meeting your current job demands’ had a residual correlation of 0.37 (RA), 0.36 (axSpA), 0.45 (OA) and 0.50 (FM), where values above 0.12 would be considered indicative of local item dependency. It was this type of grouping that enabled the making of two parallel forms in each condition, which gave adequate fit to the model, so confirming construct (structural validity). In summary, the WALS satisfied the Rasch model requirements when implemented in a bi-actor equivalent solution. The amount of variance discarded was small, giving confidence that the scale was unidimensional (albeit with a slightly inflated reliability at the item level).

**References**

1. Rasch G. *Probabilistic Models for Some Intelligence and Attainment Tests*. Chicago: The University of Chicago Press, 1980.]
2. Gustafsson JE. “Testing and obtaining fit of data to the Rasch model.” British Journal of Mathematical and Statistical Psychology 1980; 33(2), 205–233
3. Teresi JA, Kleinman M, Ocepek-Welikson K. Modern psychometric methods for detection of differential item functioning: application to cognitive assessment measures. *Stat Med* 2000; 19: 1651-1683.
4. [Kang, H.-A.](https://www.scopus.com/authid/detail.uri?authorId=57202316196&amp;eid=2-s2.0-85043310889) [Su, Y.-H.](https://www.scopus.com/authid/detail.uri?authorId=56329628300&amp;eid=2-s2.0-85043310889) [Chang, H.-H.](https://www.scopus.com/authid/detail.uri?authorId=7407524642&amp;eid=2-s2.0-85043310889) A note on monotonicity of item response functions for ordered polytomous item response theory models. [British Journal of Mathematical and Statistical Psychology](https://www.scopus.com/sourceid/12773?origin=recordpage) 2018; 71:(3),523-535
5. Rost J. An Unconditional Likelihood Ratio for Testing Item Homogeneity in the Rasch Model. Education Research and Perspectives 1982; 9(1): 7-17
6. Wilson M. Detecting and interpreting local item dependence using a family of Rasch models. Applied Psychological Measurement 1988; 12:353-364.
7. Smith EV, Jr. Detecting and evaluating the impact of multidimensionality using item fit statistics and principal component analysis of residuals. Journal of applied measurement 2002; 3(2):205-231
8. Christensen KB, Makransky G, Horton M. Critical Values for Yen's Q3: Identification of Local Dependence in the Rasch Model Using Residual Correlations. Applied psychological measurement. 2017;41(3):178-94.
9. Wainer H, Kiely G. Item clusters and computer adaptive testing: A case for testlets. *J Educ Measurement* 1987; 24:185-202.
10. Quinn HO. Bifactor models, explained common variance (ecv), and the usefulness of scores from unidimensional item response theory analyses. University of North Carolina at Chapel Hill, 2014.
11. Bland JM, Altman DG. Statistics Notes. Cronbach’s alpha. BMJ 1997: 314(7080): 572.
12. Andrich D. The Polytomous Rasch Model and the Equating of Two Instruments. In: Christensen KB, Kreiner S, Mesbah M, editors. Rasch Models in Health. London, UK: ILSTE Ltd; 2013. p. 164-96.
13. Andrich D, Sheridan BS and Luo G. RUMM2030: an MS Windows computer program for the analysis of data according to Rasch unidimensional models for measurement. Perth, Western Australia: RUMM Laboratory, 2015
14. Andrich D. Components of variance of scales with a bifactor subscale structure from two calculations of alpha. Education Measurement: Issue and Practice 2016; 35:25-30.
15. [Hagquist C](https://www.ncbi.nlm.nih.gov/pubmed/?term=Hagquist%20C%5BAuthor%5D&cauthor=true&cauthor_uid=28927468), [Andrich D](https://www.ncbi.nlm.nih.gov/pubmed/?term=Andrich%20D%5BAuthor%5D&cauthor=true&cauthor_uid=28927468)**.** Recent advances in analysis of differential item functioning in health research using the Rasch model. [Health Qual Life Outcomes.](https://www.ncbi.nlm.nih.gov/pubmed/28927468) 2017; 19;15(1):181.
16. Rouquette A**,** Hardouin J-B, Vanhaesebrouck A, Se´bille V, Coste J. Differential Item Functioning (DIF) in composite health measurement scale: Recommendations for characterizing DIF with meaningful consequences within the Rasch model framework. PLoS ONE 2019; 14 (4): e0215073.

**Supplementary Table S1: Hierarchical analytical structure for achieving ft of the Workplace Activity Limitations Scale to the Rasch model.**

| **Level** | **Nature** | **Adjustments** | **Reporting** | | |
| --- | --- | --- | --- | --- | --- |
|  |  |  | Chi-Square | ECV  ≥0.9 | Latent  Correlation  ≥0.9 |
| 1 | Item-based | None | Interaction | No | No |
| 2 | Item-Based | Clusters for Local Item Dependency | Interaction | Yes | No |
| 3 | Domain-based | On existing sub-scales >2 | Interaction | Yes | No |
| 4 | Parallel Form | On existing sub-scales <=2 or  2 LD patterns or conceptual groups | Conditional | Yes | Yes |
| 5 | Parallel Form | On alternative Items | Conditional | Yes | Yes |
| 6 | Item Deletion | On all original items  Repeat Levels 1-5 | Interaction | No | No |
| 7 | Mokken Scaling | On items if Unidimensional. Loevinger's coefficient H ≥0.4-moderate | No | No | No |
| 8 | Fail | No valid ordinal scale | No | No | No |

Key: ECV = Explained Common Variance. Interaction = Chi-Square Interaction fit statistic; Conditional = Conditional Chi-Square test of fit; Latent correlation is that between two items sets that are deemed to be parallel forms.

**Supplementary Figure S1: Recruitment Flowchart Phase 2**

1359 referred from PICs

n = 480 not included (35.3%)

407 = No reply (84.8%)

40 = Received after recruitment closed for condition group (8.3%)

20 = Ineligible (e.g., long term sick leave; retired; incorrect diagnosis (4.2%))

10 = Withdrawn (2%)

3 = Administration errors (e.g., wrong address (0.06%))

831 participants

RA

n = 299

axSpA

n = 206

OA

n = 213

FM

n = 161

FM

n = 156

OA

n = 176

axSpA

n = 202

RA

n = 297

879 returned questionnaires

n= 48 Excluded (5.5%)

RA: n = 2* (Incorrect diagnosis n=2)

axSpA: n = 4* (Incorrect diagnosis n=3; retired n=1)

OA: n = 37* (Incorrect diagnosis n=7; not Lower limb OA n=30)

FM: n = 5* (Incorrect diagnosis n=4; unemployed n=1)

Key: *Incorrect diagnosis based on participant self-report in questionnaire

Key: PICs – Patient Identification Centres; RA = rheumatoid arthritis; axSpA = axial spondyloarthritis; OA = osteoarthritis; FM = fibromyalgia.

Volunteers

n = 16 (1.90%)

Secondary Care NHS Trusts

n = 696 (83.80%)

Community NHS

n = 119 (14.30%)

**Supplementary Table S2**: **Phase 1: relevance of Workplace Activity Limitations Scale items (n = 48)**

| **Item no.** | **Item** | **RA**  **n=12** | **axSpA**  **n=10** | **OA**  **n=13** | **FM**  **n=13** | **Chi square** | **df** | **p** |
| --- | --- | --- | --- | --- | --- | --- | --- | --- |
| 1 | Get to/ from work | 5.00 (4.00 – 5.00) | 5.00 (5.00 – 5.00) | 5.00 (4.00 – 5.00) | 5.00 (4.50 – 5.00) | 12.15 | 9 | 0.21 |
| 2 | Get around work | 5.00 (4.00 – 5.00) | 5.00 (4.00 – 5.00) | 5.00 (4.00 – 5.00) | 5.00 (4.50- 5.00) | 4.24 | 6 | 0.64 |
| 3 | Sit for long periods | 5.00 (4.00 – 5.00) | 5.00 (4.75 – 5.00) | 5.00 (4.00 – 5.00) | 5.00 (4.00 – 5.00) | 5.26 | 6 | 0.51 |
| 4 | Stand for long periods | 5.00 (4.00 – 5.00) | 5.00 (3.75 – 5.00) | 5.00 (4.00 – 5.00) | 5.00 (4.00 – 5.00) | 10.14 | 9 | 0.34 |
| 5 | Lift, carry, move objects | 5.00 (4.00 – 5.00) | 5.00 (4.00 – 5.00) | 5.00 (4.00 – 5.00) | 5.00 (4.00 – 5.00) | 8.17 | 9 | 0.52 |
| 6 | Work with hands | 5.00 (4.00 – 5.00) | 5.00 (4.75 – 5.00) | 5.00 (4.00 – 5.00) | 5.00 (4.00 – 5.00) | 9.65 | 9 | 0.38 |
| 7 | Crouch, bend, kneel | 5.00 (4.00 – 5.00) | 5.00 (4.00 – 5.00) | 5.00 (4.00 – 5.00) | 5.00 (4.00 – 5.00) | 7.48 | 9 | 0.59 |
| 8 | Reach | 5.00 (4.00 – 5.00) | 5.00 (4.50 – 5.00) | 5.00 (4.00 – 5.00) | 5.00 (4.00 – 5.00) | 10.85 | 9 | 0.29 |
| 9 | Manage hours of work | 5.00 (4.00 – 5.00) | 5.00 (4.75 – 5.00) | 5.00 (4.00 – 5.00) | 5.00 (4.50 – 5.00) | 9.47 | 9 | 0.40 |
| 10 | Manage pace of work | 5.00 (4.00 – 5.00) | 5.00 (5.00 – 5.00) | 5.00 (4.00 – 5.00) | 5.00 (4.00 – 5.00) | 10.36 | 9 | 0.32 |
| 11 | Manage job demands | 5.00 (4.00 – 5.00) | 5.00 (5.00 – 5.00) | 5.00 (4.00 – 5.00) | 5.00 (4.50 – 5.00) | 11.99 | 9 | 0.21 |
| 12 | Concentrate at work | 5.00 (4.00 – 5.00) | 5.00 (4.75 – 5.00) | 5.00 (4.00 – 5.00) | 5.00 (4.50 – 5.00) | 7.92 | 9 | 0.54 |

Key: 1 = not at all relevant; 5 = extremely relevant. RA = rheumatoid arthritis; axSpA = axial spondyloarthritis; OA = osteoarthritis; FM = fibromyalgia.

No significant differences between groups.

**Supplementary Table S3: Content validity of Workplace Activity Limitations Scale linked to the ICF Core Set for Vocational Rehabilitation (Activities and Participation domain).**

| **Work Activity Limitations Scale items** | | | | | | | | | | | | | |
| --- | --- | --- | --- | --- | --- | --- | --- | --- | --- | --- | --- | --- | --- |
| **ICF Vocational Rehabilitation Core Set Activities and Participation domain** | | 1  To/ from work | 2  Get around work | 3  Sit | 4  Stand | 5  Lift,  carry | 6  Work with hands | 7  Crouch, bend, kneel | 8  Reach | 9  Manage hours | 10  Manage pace | 11  Job demands | 12  Concen-tration |
| 1 | d155 Acquiring skills |  |  |  |  |  |  |  |  |  |  |  |  |
| 2 | d160 Focusing attention |  |  |  |  |  |  |  |  |  |  |  |  |
| 3 | d163 Thinking |  |  |  |  |  |  |  |  |  |  |  |  |
| 4 | d166 Reading |  |  |  |  |  |  |  |  |  |  |  |  |
| 5 | d170 Writing |  |  |  |  |  |  |  |  |  |  |  |  |
| 6 | d172 Calculating |  |  |  |  |  |  |  |  |  |  |  |  |
| 7 | d175 Solving problems |  |  |  |  |  |  |  |  |  |  |  |  |
| 8 | d177 Making decisions |  |  |  |  |  |  |  |  |  |  |  |  |
| 9 | d210 Undertaking a single task |  |  |  |  |  |  |  |  |  |  |  |  |
| 10 | d220 Undertaking multiple tasks |  |  |  |  |  |  |  |  |  |  |  |  |
| 11 | d230 Carrying out daily routine |  |  |  |  |  |  |  |  |  |  |  |  |
| 12 | d240 Handling stress and other psychological demands |  |  |  |  |  |  |  |  |  |  |  |  |
| 13 | d310 Communicating with - receiving - spoken messages |  |  |  |  |  |  |  |  |  |  |  |  |
| 14 | d315 Communicating with - receiving - nonverbal messages |  |  |  |  |  |  |  |  |  |  |  |  |
| 15 | d350 Conversation |  |  |  |  |  |  |  |  |  |  |  |  |
| 16 | d360 Using communication devices and techniques |  |  |  |  |  |  |  |  |  |  |  |  |
| 17 | d410 Changing basic body position |  |  |  |  |  |  |  |  |  |  |  |  |
| 18 | d415 Maintaining a body position |  |  |  |  |  |  |  |  |  |  |  |  |
| 19 | d430 Lifting and carrying objects |  |  |  |  |  |  |  |  |  |  |  |  |
| 20 | d440 Fine hand use |  |  |  |  |  |  |  |  |  |  |  |  |
| 21 | d445 Hand and arm use |  |  |  |  |  |  |  |  |  |  |  |  |
| 22 | d450 Walking |  |  |  |  |  |  |  |  |  |  |  |  |
| 23 | d455 Moving around |  |  |  |  |  |  |  |  |  |  |  |  |
| 24 | d465 Moving around using equipment |  |  |  |  |  |  |  |  |  |  |  |  |
| 25 | d470 Using transportation |  |  |  |  |  |  |  |  |  |  |  |  |
| 26 | d475 Driving |  |  |  |  |  |  |  |  |  |  |  |  |
| 27 | d530 Toileting |  |  |  |  |  |  |  |  |  |  |  |  |
| 28 | d540 Dressing |  |  |  |  |  |  |  |  |  |  |  |  |
| 29 | d570 Looking after one’s health |  |  |  |  |  |  |  |  |  |  |  |  |
| 30 | d710 Basic interpersonal interactions |  |  |  |  |  |  |  |  |  |  |  |  |
| 31 | d720 Complex interpersonal interactions |  |  |  |  |  |  |  |  |  |  |  |  |
| 32 | d740 Formal relationships |  |  |  |  |  |  |  |  |  |  |  |  |
| 33 | d820 School education |  |  |  |  |  |  |  |  |  |  |  |  |
| 34 | d825 Vocational training |  |  |  |  |  |  |  |  |  |  |  |  |
| 35 | d830 Higher education |  |  |  |  |  |  |  |  |  |  |  |  |
| 36 | d840 Apprenticeship (work preparation |  |  |  |  |  |  |  |  |  |  |  |  |
| 37 | d845 Acquiring, keeping, and terminating a job |  |  |  |  |  |  |  |  |  |  |  |  |
| 38 | d850 Remunerative employment |  |  |  |  |  |  |  |  |  |  |  |  |
| 39 | d855 Non-remunerative employment |  |  |  |  |  |  |  |  |  |  |  |  |
| 40 | d870 Economic self-sufficiency |  |  |  |  |  |  |  |  |  |  |  |  |

Note: Item 11: “Meeting your current job demands” predominantly relates to Chapter 2: General Tasks and Demands” in the ICF, which states that “These items can be used in conjunction with more specific tasks or actions to identify the underlying features of the execution of tasks in different circumstances.” Codes shown in light grey may be relevant depending on the nature of the person’s job and how the respondent interprets this WALS item.

**Supplementary Table S4: Frequency of “not applicable” and missing items in the Workplace Activity Limitations Scale.**

| **WALS items** | **RA (n=297)**  **n (%)** | | axSpA **(n=202)**  **n (%)** | | **OA (n=176)**  **n (%)** | | **FM (n=156)**  **n (%)** | |
| --- | --- | --- | --- | --- | --- | --- | --- | --- |
|  | Not applicable | Missing | Not applicable | Missing | Not applicable | Missing | Not applicable | Missing |
| 1. Get to/from work | 22 (7.40) | 2 (0.70) | 9 (4.50) | 2 (1.00) | 10 (5.70) | 0 (0) | 7 (4.50) | 0 |
| 2. Get around at work | 17 (5.70) | 3 (1.00) | 6 (3.00) | 2 (1.00) | 6 (3.40) | 0 (0( | 9 (5.80) | 0 |
| 3. Sit for long periods | 47 (15.80) | 1 (0.30) | 21 (10.40) | 2 (1.00) | 19 (10.80) | 0 (0) | 18 (11.50) | 0 |
| 4. Stand for long periods | 57 (19.20) | 1 (0.30) | 23 (11.40) | 2 (1.00) | 17 (9.70) | 2 (1.10) | 20 (12.80) | 0 |
| 5. Lift, carry, move objects | 29 (9.80) | 0 (0) | 15 (7.40) | 2 (1.00) | 17 (9.70) | 1 (0.60) | 18 (11.50) | 0 |
| 6. Work with hands | 7 (2.40) | 3 (1.00) | 3 (1.50) | 2 (1.00) | 7 (4.00) | 2 (1.10) | 1 (0.60) | 0 |
| 7. Crouch, bend, kneel | 30 (10.10) | 3 (1.00) | 20 (9.90) | 2 (1.00) | 12 (6.80) | 2 (1.10) | 21 (13.50) | 0 |
| 8. Reach | 20 (6.70) | 3 (1.00) | 12 (5.90) | 2 (1.00) | 11 (6.30) | 3 (1.70) | 16 (10.30) | 0 |
| 9. Manage hours of work | 30 (10.10) | 3 (1.00) | 15 (7.40) | 2 (1.00) | 17 (9.70) | 3 (1.70) | 7 (4.50) | 0 |
| 10. Manage pace of work | 14 (4.70) | 2 (0.70) | 9 (4.50) | 2 (1.00) | 11 (6.30) | 2 (1.10) | 4 (2.60) | 0 |
| 11. Meeting job demands | 10 (3.40) | 2 (0.70) | 4 (2.00) | 2 (1.00) | 6 (3.40) | 3 (1.70) | 3 (1.90) | 0 |
| 12. Concentrating at work | 6 (2.00) | 2 (0.70) | 4 (2.00) | 2 (1.00) | 9 (5.10) | 2 (1.10) | 3 (1.90) | 0 |

Key: WALS = Work Activity Limitations Scale; RA = rheumatoid arthritis; axSpA = axial spondyloarthritis; OA = osteoarthritis; FM = fibromyalgia

**Supplementary Table S5. Raw score to interval scale transformation of the Workplace Activity Limitations Scale.**

| **WALS Raw Score** | **WALS Interval Score** |
| --- | --- |
| 0 | 0 |
| 1 | 2.90 |
| 2 | 5.00 |
| 3 | 6.50 |
| 4 | 7.70 |
| 5 | 8.70 |
| 6 | 9.60 |
| 7 | 10.40 |
| 8 | 11.20 |
| 9 | 11.90 |
| 10 | 12.50 |
| 11 | 13.10 |
| 12 | 13.60 |
| 13 | 14.10 |
| 14 | 14.60 |
| 15 | 15.10 |
| 16 | 15.50 |
| 17 | 16.00 |
| 18 | 16.40 |
| 19 | 16.90 |
| 20 | 17.40 |
| 21 | 17.90 |
| 22 | 18.40 |
| 23 | 18.90 |
| 24 | 19.50 |
| 25 | 20.20 |
| 26 | 20.90 |
| 27 | 21.60 |
| 28 | 22.50 |
| 29 | 23.40 |
| 30 | 24.40 |
| 31 | 25.50 |
| 32 | 26.70 |
| 33 | 28.20 |
| 34 | 30.00 |
| 35 | 32.50 |
| 36 | 36.00 |

Key: WALS: Work Activity Limitations Scale

**Supplementary Table S6: Calibration of the Workplace Activity Limitations Scale and Work Instability Scales on the Reference Metric.**

| **Raw score** | **Reference** | **WALS** | **AS-WIS** | **RA-WIS** |
| --- | --- | --- | --- | --- |
| 0 | 0.0 | 26.9 | 24.8 | 23.8 |
| 1 | 9.7 | 31.8 | 29.3 | 29.3 |
| 2 | 15.5 | 35.2 | 32.3 | 33.0 |
| 3 | 19.0 | 37.6 | 34.4 | 35.3 |
| 4 | 21.4 | 39.5 | 36.0 | 37.1 |
| 5 | 23.2 | 41.2 | 37.4 | 38.5 |
| 6 | 24.6 | 42.7 | 38.7 | 39.7 |
| 7 | 25.7 | 44.1 | 39.8 | 40.7 |
| 8 | 26.6 | 45.5 | 40.9 | 41.7 |
| 9 | 27.5 | 46.9 | 42.0 | 42.6 |
| 10 | 28.2 | 48.2 | 43.1 | 43.5 |
| 11 | 28.8 | 49.5 | 44.1 | 44.4 |
| 12 | 29.4 | 50.7 | 45.2 | 45.4 |
| 13 | 29.9 | 51.9 | 46.3 | 46.3 |
| 14 | 30.4 | 53.1 | 47.5 | 47.4 |
| 15 | 30.8 | 54.2 | 48.8 | 48.6 |
| 16 | 31.3 | 55.4 | 50.2 | 50.0 |
| 17 | 31.7 | 56.5 | 51.8 | 51.6 |
| 18 | 32.0 | 57.7 | 53.9 | 53.5 |
| 19 | 32.4 | 58.8 | 57.0 | 55.8 |
| 20 | 32.7 | 60.0 | 61.6 | 58.6 |
| 21 | 33.1 | 61.2 |  | 62.0 |
| 22 | 33.4 | 62.4 |  | 67.0 |
| 23 | 33.7 | 63.6 |  | 74.0 |
| 24 | 34.0 | 64.9 |  |  |
| 25 | 34.2 | 66.2 |  |  |
| 26 | 34.5 | 67.5 |  |  |
| 27 | 34.8 | 68.9 |  |  |
| 28 | 35.0 | 70.4 |  |  |
| 29 | 35.3 | 71.9 |  |  |
| 30 | 35.5 | 73.4 |  |  |
| 31 | 35.8 | 75.1 |  |  |
| 32 | 36.0 | 76.9 |  |  |
| 33 | 36.3 | 78.9 |  |  |
| 34 | 36.5 | 81.4 |  |  |
| 35 | 36.7 | 85.0 |  |  |
| 36 | 37.0 | 90.2 |  |  |
| 37 | 37.2 |  |  |  |
| 38 | 37.4 |  |  |  |
| 39 | 37.6 |  |  |  |
| 40 | 37.8 |  |  |  |
| 41 | 38.0 |  |  |  |
| 42 | 38.2 |  |  |  |
| 43 | 38.4 |  |  |  |
| 44 | 38.7 |  |  |  |
| 45 | 38.9 |  |  |  |
| 46 | 39.1 |  |  |  |
| 47 | 39.3 |  |  |  |
| 48 | 39.5 |  |  |  |
| 49 | 39.6 |  |  |  |
| 50 | 39.8 |  |  |  |
| 51 | 40.0 |  |  |  |
| 52 | 40.2 |  |  |  |
| 53 | 40.4 |  |  |  |
| 54 | 40.6 |  |  |  |
| 55 | 40.8 |  |  |  |
| 56 | 41.0 |  |  |  |
| 57 | 41.2 |  |  |  |
| 58 | 41.4 |  |  |  |
| 59 | 41.5 |  |  |  |
| 60 | 41.7 |  |  |  |
| 61 | 41.9 |  |  |  |
| 62 | 42.1 |  |  |  |
| 63 | 42.3 |  |  |  |
| 64 | 42.4 |  |  |  |
| 65 | 42.6 |  |  |  |
| 66 | 42.8 |  |  |  |
| 67 | 43.0 |  |  |  |
| 68 | 43.2 |  |  |  |
| 69 | 43.3 |  |  |  |
| 70 | 43.5 |  |  |  |
| 71 | 43.7 |  |  |  |
| 72 | 43.8 |  |  |  |
| 73 | 44.0 |  |  |  |
| 74 | 44.2 |  |  |  |
| 75 | 44.4 |  |  |  |
| 76 | 44.5 |  |  |  |
| 77 | 44.7 |  |  |  |
| 78 | 44.9 |  |  |  |
| 79 | 45.0 |  |  |  |
| 80 | 45.2 |  |  |  |
| 81 | 45.4 |  |  |  |
| 82 | 45.5 |  |  |  |
| 83 | 45.7 |  |  |  |
| 84 | 45.9 |  |  |  |
| 85 | 46.0 |  |  |  |
| 86 | 46.2 |  |  |  |
| 87 | 46.3 |  |  |  |
| 88 | 46.5 |  |  |  |
| 89 | 46.7 |  |  |  |
| 90 | 46.8 |  |  |  |
| 91 | 47.0 |  |  |  |
| 92 | 47.2 |  |  |  |
| 93 | 47.3 |  |  |  |
| 94 | 47.5 |  |  |  |
| 95 | 47.7 |  |  |  |
| 96 | 47.8 |  |  |  |
| 97 | 48.0 |  |  |  |
| 98 | 48.1 |  |  |  |
| 99 | 48.3 |  |  |  |
| 100 | 48.4 |  |  |  |
| 101 | 48.6 |  |  |  |
| 102 | 48.8 |  |  |  |
| 103 | 48.9 |  |  |  |
| 104 | 49.1 |  |  |  |
| 105 | 49.2 |  |  |  |
| 106 | 49.4 |  |  |  |
| 107 | 49.5 |  |  |  |
| 108 | 49.7 |  |  |  |
| 109 | 49.9 |  |  |  |
| 110 | 50.0 |  |  |  |
| 111 | 50.2 |  |  |  |
| 112 | 50.3 |  |  |  |
| 113 | 50.5 |  |  |  |
| 114 | 50.6 |  |  |  |
| 115 | 50.8 |  |  |  |
| 116 | 51.0 |  |  |  |
| 117 | 51.1 |  |  |  |
| 118 | 51.3 |  |  |  |
| 119 | 51.4 |  |  |  |
| 120 | 51.6 |  |  |  |
| 121 | 51.8 |  |  |  |
| 122 | 51.9 |  |  |  |
| 123 | 52.1 |  |  |  |
| 124 | 52.3 |  |  |  |
| 125 | 52.4 |  |  |  |
| 126 | 52.6 |  |  |  |
| 127 | 52.8 |  |  |  |
| 128 | 52.9 |  |  |  |
| 129 | 53.1 |  |  |  |
| 130 | 53.3 |  |  |  |
| 131 | 53.5 |  |  |  |
| 132 | 53.6 |  |  |  |
| 133 | 53.8 |  |  |  |
| 134 | 54.0 |  |  |  |
| 135 | 54.2 |  |  |  |
| 136 | 54.4 |  |  |  |
| 137 | 54.6 |  |  |  |
| 138 | 54.7 |  |  |  |
| 139 | 54.9 |  |  |  |
| 140 | 55.1 |  |  |  |
| 141 | 55.3 |  |  |  |
| 142 | 55.5 |  |  |  |
| 143 | 55.7 |  |  |  |
| 144 | 55.9 |  |  |  |
| 145 | 56.1 |  |  |  |
| 146 | 56.3 |  |  |  |
| 147 | 56.6 |  |  |  |
| 148 | 56.8 |  |  |  |
| 149 | 57.0 |  |  |  |
| 150 | 57.2 |  |  |  |
| 151 | 57.4 |  |  |  |
| 152 | 57.7 |  |  |  |
| 153 | 57.9 |  |  |  |
| 154 | 58.2 |  |  |  |
| 155 | 58.4 |  |  |  |
| 156 | 58.7 |  |  |  |
| 157 | 58.9 |  |  |  |
| 158 | 59.2 |  |  |  |
| 159 | 59.5 |  |  |  |
| 160 | 59.7 |  |  |  |
| 161 | 60.0 |  |  |  |
| 162 | 60.3 |  |  |  |
| 163 | 60.6 |  |  |  |
| 164 | 61.0 |  |  |  |
| 165 | 61.3 |  |  |  |
| 166 | 61.6 |  |  |  |
| 167 | 62.0 |  |  |  |
| 168 | 62.4 |  |  |  |
| 169 | 62.7 |  |  |  |
| 170 | 63.1 |  |  |  |
| 171 | 63.6 |  |  |  |
| 172 | 64.0 |  |  |  |
| 173 | 64.5 |  |  |  |
| 174 | 65.0 |  |  |  |
| 175 | 65.5 |  |  |  |
| 176 | 66.1 |  |  |  |
| 177 | 66.7 |  |  |  |
| 178 | 67.3 |  |  |  |
| 179 | 68.0 |  |  |  |
| 180 | 68.7 |  |  |  |
| 181 | 69.4 |  |  |  |
| 182 | 70.2 |  |  |  |
| 183 | 71.1 |  |  |  |
| 184 | 72.0 |  |  |  |
| 185 | 73.0 |  |  |  |
| 186 | 74.0 |  |  |  |
| 187 | 75.2 |  |  |  |
| 188 | 76.4 |  |  |  |
| 189 | 77.7 |  |  |  |
| 190 | 79.3 |  |  |  |
| 191 | 81.1 |  |  |  |
| 192 | 83.4 |  |  |  |
| 193 | 86.5 |  |  |  |
| 194 | 91.6 |  |  |  |
| 195 | 100.0 |  |  |  |

Key: WALS = Work Activity Limitations Scale; WIS = Work Instability Scale; RA = rheumatoid arthritis; AS = ankylosing spondyloarthritis

**Supplementary Table S7: Discriminant validity of the Workplace Activity Limitations Scale.**

| **Perceived health status:** | **Very poor/ poor** | **Fair** | **Good/ very good** | **Kruskal-Wallis H** | **df** | **p** |
| --- | --- | --- | --- | --- | --- | --- |
| RA (n = 294) | 15.00 (11.00 – 19.50)  n=45 | 11.00 (8.00 – 14.00)  n = 133 | 5.00 (3.00 – 8.00)  n = 116 | 104.72 | 2 | <0.001 |
| axSpA (n=199) | 12.00 (4.50 – 15.00)  n = 21 | 9.00 (4.00 – 12.00)  n = 78 | 4.00 (2.00 – 9.00)  n = 100 | 24.47 | 3 | <0.001 |
| OA (n=173) | 15.00 (12.00 – 18.50)  n = 37 | 10.00 (6.00 – 13.00)  n = 95 | 6.00 (3.00 – 8.50)  n = 41 | 50.28 | 2 | <0.001 |
| FM (n=156) | 1700. (15.00 -21.00)  n = 83 | 14.00 (10.00 – 16.00)  n = 63 | 11.45 (4.75 – 14.25)  n = 10 | 30.94 | 2 | <0.001 |

Key: RA = rheumatoid arthritis; axSpA = axial spondyloarthritis; OA = osteoarthritis; FM = fibromyalgia; WALS = Work Activity Limitations Scale

WALS: higher scores indicate greater work limitations.

**Supplementary Table S8: Workplace Activity Limitations Scale item test-retest reliability (quadratic weighted kappa) in RA, axSpA, OA and FM.**

| **WALS items** | **RA (n=136) ^1^**  **n (%)** | **axSpA (n=88) ^1^**  **n (%)** | **OA (n=78) ^1^**  **n (%)** | **FM (n=54) ^1^**  **n (%)** |
| --- | --- | --- | --- | --- |
| 1. Get to/from work | 0.69 | 0.64 | 0.73 | 0.45 |
| 2. Get around at work | 0.66 | 0.50 | 0.51 | 0.67 |
| 3. Sit for long periods | 0.77 | 0.56 | 0.58 | 0.73 |
| 4. Stand for long periods | 0.66 | 0.65 | 0.57 | 0.59 |
| 5. Lift, carry, move objects | 0.60 | 0.57 | 0.66 | 0.63 |
| 6. Work with hands | 0.61 | 0.68 | 0.75 | 0.56 |
| 7. Crouch, bend, kneel | 0.67 | 0.57 | 0.59 | 0.52 |
| 8. Reach | 0.69 | 0.55 | 0.61 | 0.49 |
| 9. Manage hours of work | 0.55 | 0.51 | 0.44 | 0.61 |
| 10. Manage pace of work | 0.64 | 0.50 | 0.55 | 0.47 |
| 11. Meeting job demands | 0.68 | 0.68 | 0.69 | 0.60 |
| 12. Concentrating at work | 0.68 | 0.73 | 0.64 | 0.49 |

Key: ^1^ Participants reporting” the same” health status at T1 and T2; RA = rheumatoid arthritis; axSpA = axial spondyloarthritis; OA = osteoarthritis; FM = fibromyalgia.
